# Polyphasic, Including MALDI-TOF MS, Evaluation of Freeze-Drying Long-Term Preservation on *Aspergillus* (Section *Nigri*) Strains

**DOI:** 10.3390/microorganisms7090291

**Published:** 2019-08-25

**Authors:** Rodrigo Rodriguez, Carla Santos, Marta F. Simões, Célia Soares, Cledir Santos, Nelson Lima

**Affiliations:** 1 CEB-Centre of Biological Engineering, Micoteca da Universidade do Minho, University of Minho, 4710-057 Braga, Portugal; 2 State Key Laboratory of Lunar and Planetary Sciences, Space Science Institute. Macau University of Science and Technology, Avenida Wai Long, Taipa, Macau SAR 999078, China; 3 Department of Chemical Sciences and Natural Resources, BIOREN-UFRO Scientific and Technological Bioresource Nucleus, Universidad de La Frontera, Temuco 4811-230, Chile

**Keywords:** freeze-drying, accelerated ageing, mass spectrometry, DNA fingerprinting, biotechnological potential

## Abstract

This study aims to evaluate the effect of freeze-drying and long-term storage on the biotechnological potential of *Aspergillus* section *Nigri* strains. Twelve selected strains were freeze-dried and aged by accelerated storage, at 37 °C in the dark, for 2 and 4 weeks. To assess possible changes as a consequence of the ageing in the freeze-drying ampoules, morphological characteristics, mycotoxins and enzymes production, matrix-assisted laser desorption/ionisation time-of-flight mass spectrometry (MALTI-TOF MS) spectra, and M13 phage probe fingerprinting were used as part of a polyphasic approach. Phenotypical changes were observed; nevertheless, they did not substantially affect the potential biotechnological use of these strains. The activity of hydrolytic enzymes (protease, carboxymethylcellulase, xylanase, pectinase and mannanase) was maintained or increased after freeze-drying. MALDI-TOF MS data originated spectra that grouped, for the majority of samples, according to strain independently of preservation time point. M13 profiles revealed the presence of some genetic polymorphisms after preservation. However, the three studied times still clustered for more than 50% of strains. Our results show that the studied strains maintain their biotechnological potential after preservation, with minimal phenotypic alterations. These findings provide evidence that freeze-drying preservation is a suitable option to preserve biotechnologically relevant aspergilli strains from section *Nigri*, and one should consider that the observed effects might be species/strain-dependent.

## 1. Introduction

Environmental fungal strains can be used in (1) research, (2) diagnostic laboratories (as reference strains), (3) academic laboratories, (4) industrial biotechnology processes as well as (5) environmental biotechnology (e.g., bioremediation) [1,2]. Maintaining and preserving relevant strains is essential for research and commercial exploitation of the fungal biodiversity [3].

The Aspergilli are among the most abundant fungi on earth [4]. They can synthesise multiple metabolites, which allow them to adapt to the variety of environments they inhabit. Some of these primary and secondary metabolites have been commercially exploited; generating several *Aspergillus*-related products and patents including antibiotics, anti-tumoral and anti-fungal agents as well as other compounds with a medical application, such as the cholesterol-lowering drug lovastatin [5,6,7,8,9]. Additionally, Aspergilli strains are involved in many modern industrial processes including the production of hydrolytic enzymes (e.g., pectinases) [10] and bulk chemicals (e.g., citric acid) but have also been used for centuries in Asia for the production of fermented foods, such as sake, soy sauce and miso [11]. On the other hand, some *Aspergillus* strains are pathogens to humans and animals, being the causing agent of, e.g., aspergillomas and allergic bronchopulmonary aspergillosis in the case of *A. fumigatus*, and mycotoxicosis related with secondary metabolites produced by *A. carbonarius*, *A. clavatus*, *A. flavus*, *A. niger*, *A. ochraceus*, *A. parasiticus* and *A. terreus* [12,13]. In agriculture, *A. flavus*, *A. parasiticus*, *A. niger*, *A. ochraceus* and *A. carbonarius* can cause deterioration of stored crops and are opportunistic pathogens of field crops [14].

Preservation aims to find the storage conditions that minimise the number of generations from the initial isolate and decrease the metabolic activity of the cell to a minimal one [15]. Since fungi are such a diverse group, several different methods of cultivation and preservation are required to ensure that viability, and morphological, physiological and genetic characteristics of the cultures are conserved over time [16]. There are several techniques available for the preservation of filamentous fungi; nevertheless, freeze-drying is the most reliable method for long-term preservation [17]. Despite this, it is important to mention that no preservation method fits all the species and that no method guarantees total physiological and genetic stability of an isolate [1,18,19,20].

Recently, consistent identification and characterisation of species of filamentous fungi have been achieved employing the so-called polyphasic approach [1,12,14,21]. The polyphasic approach combines the use of different techniques, such as morphology, biochemical and molecular biology tools and more recently, mass spectrometry (particularly matrix-assisted laser desorption/ionisation time-of-flight mass spectrometry (MALDI-TOF MS). Furthermore, mass spectrometry based microbial spectral analysis has been used as a key step in the polyphasic identification of filamentous fungi from different fields [12,14,22,23,24,25,26,27,28].

The time of storage after preservation at which a given percentage of the original population remains viable and unchanged is called shelf-life. Therefore, it is desirable to know for how long the cells will remain viable and how viability will be influenced by the different preservation methods. Because of the difficulty in testing the effects of long-term storage on microbial viability, storage based on accelerated conditions that mimic the effect of long-term preservation has been previously used to promote the process of cell ageing [29,30]. It has been shown that accelerated storage of liquid-dried bacterial cultures for 2 weeks at 37 °C can simulate 20 years of storage at 5 °C [29]. The process of accelerated storage has shown to be a useful tool to evaluate the efficiency of different preservation methods as well as to predict the behaviour of preserved microbiological cells after long-term storage [29,30].

Although several studies have evaluated the use of different methods of preservation on *Aspergillus* strains viability [19,31,32,33,34,35], to our knowledge, none have assessed possible effects on a wide range of phenotypical characteristics after preservation in this genus. The biotechnological relevant species—*Aspergillus niger*—was also included in this selection as a model organism since it has been used as a cell factory for the production of a wide range of metabolites, such as organic acids, antibiotics, extracellular enzymes, as well as native and recombinant proteins. The present work aimed to apply a polyphasic approach (morphology, molecular markers, mycotoxins, enzyme secretion and MALDI-TOF MS spectral profiling) to characterise the effects of freeze-drying and accelerated storage on the biotechnological potential of 12 strains belonging to different species within *Aspergillus* section *Nigri*.

## 2. Materials and Methods

### 2.1. Fungal Strains

Twelve strains belonging to *Aspergillus* section *Nigri* were selected and supplied by the Filamentous Fungal Culture Collection Micoteca da Universidade do Minho (MUM, Braga, Portugal) (Table 1). All samples were initially grown in malt extract agar (MEA, malt extract 20 g/L, mycological peptone 1 g/L, glucose 20 g/L, agar 20 g/L) and incubated in the dark at 25 °C for 7 days, for further analyses.

### 2.2. Macro and Micro-Morphological Evaluation

For macro-morphological analysis, the selected strains were grown on: MEA, potato dextrose agar (PDA, potato infusion 200 g/L, dextrose 20 g/L, agar 15 g/L), Czapek agar (CZ, sucrose 30 g/L, K_2_HPO_4_ 1 g/L, KCl 0.5 g/L, NaNO_3_ 2 g/L, MgSO_4_ 0.5 g/L, FeSO_4_ 0.01 g/L, agar 15 g/L) and Czapek agar with yeast extract (CYA, sucrose 30 g/L, yeast extract 5 g/L, K_2_HPO_4_ 1 g/L, NaNO_3_ 0.3 g/L, KCl 0.05 g/L, MgSO_4_ 0.05 g/L, FeSO_4_ 0.001 g/L, ZnSO_4_ 0.001 g/L, CuSO_4_ 0.0005 g/L, agar 15 g/L). Each plate was inoculated at 3 equidistant points and incubated in the dark, at 25 °C for 7 days.

For microscopic and stereomicroscopic analyses, the strains were grown for 3 or 4 days on MEA. To have a clear image comparison of the size of the conidial head, a Leica MZ12.5 stereomicroscope (Leica Microsystems GmbH, Wetzlar, Germany) was used. The fungal characterisation was carried out using a Leica DM R light microscope (Leica Microsystems GmbH, Wetzlar, Germany). Samples were mounted on a slide, abundantly washed with ethanol (96%) to remove excessive spores and stained with a lactophenol blue solution.

### 2.3. Proteolytic Activity Determination

This assay was done by evaluating the activity of proteases through substrate degradation, observed by the appearance of a transparent column beneath the fungal colony. Skim milk agar medium (SKM, glucose 10 g/L, KH_2_PO_4_ 1 g/L, KCl 0.5 g/L, MgSO_4·_7H_2_O 0.2 g/L, CaCl_2_·7H_2_O 0.1 g/L, skim milk 22.5% (w/v) 25 mL/L, agar 12 g/L) was prepared, sterilised by autoclavation at 110 °C for 15 min and distributed into Falcon tubes (5 mL per tube), leaving a flat surface on the medium. The tubes were inoculated on the top with a 12 mm disc with all the 12 selected strains at the 3 different time points (I, II and III), and were incubated at 25 °C in the dark for 7 days. The deep-clearing distance was measured in millimetres from the inoculated disc on the 7th day.

### 2.4. Polysaccharide-Hydrolytic Activity Determination

The enzymatic activity was assessed by screening the activity of a set of polysaccharide-degrading enzymes, which was performed according to the protocols used for high throughput screening of enzymes used at the Culture Collection CIRM-CF (International Centre of Microbial Resources dedicated to Filamentous Fungi) from the French National Institute for Agricultural Research (INRA, Marseille, France) [36].

The selected strains of *Aspergillus* used in this study were initially grown in MEA and incubated for 7 days at 25 °C in the dark. Spores from 7 day-old cultures were scraped with a sterile scraper and recovered into sterile 50 mL Falcon tubes using sterile NaCl 8.5 g/L solution containing Tween 80 (0.02%). The fungal cultures were grown in 250 mL-baffled flasks containing 100 mL of malt extract-glucose–yeast extract-peptone (MGYP, malt extract 3 g/L, glucose 10 g/L, yeast extract 3 g/L, peptone 5 g/L). The cultures were inoculated with 2 × 10^5^ spores/mL and incubated at 25 °C in duplicate under shaking at 150 rpm with a Certomat rotary shaker for 7 days.

Aliquots of 2 mL from each 100 mL culture were taken at different days of incubation (1, 2, 3, 5 and 7 days), and filtered with 0.45 µm polyethersulfone membrane (Vivaspin^®^ Sartorius, Aubagne, France). All the tubes containing the filtered culture media (enzymatic extracts) were kept at −20 °C until the analyses.

Polysaccharide-degrading enzyme miniaturised assays were performed using complex substrates on 96-well plates, as previously described [36]. To measure carboxymethylcellulase (CMCase), xylanase, pectinase and mannanase activities, complex substrates used in this study were carboxymethylcellulose (CMC), wheat xylan (WX), pectin of *Citrus* sp. (PC) and galactomannan (Man), respectively. Solutions (1% w/v) were prepared in sodium acetate buffer 50 mM at pH = 5.2. In microtubes, 25 µL of enzymatic extract was added to 150 µL of each substrate (200 µL of final volume per microtube). Afterwards, all microtubes were incubated at 37 °C for 2 h. Reducing sugars were quantified using the adapted dinitrosalicylic acid (DNS) method described by [37]. The enzyme activities were expressed as nanokatal/millilitre, defined as 1 nmol glucose equivalent released per millilitre of medium and per second under the assay conditions. Glucose was used to determine a standard curve. In addition, and for control, the total protein was determined by the Bradford method using bovine serum albumin as standard [38]. A miniaturised assay was performed on a 96-well plate, with a final volume of 200 µL, using 40 µL of reagent and 160 µL of the sample. After homogenisation, the absorbance was measured at 595 nm.

### 2.5. Assessment of Mycotoxins Production

For ochratoxin A (OTA) production, all *Aspergillus* strains were tested in yeast extract sucrose (YES, yeast extract 20 g/L, sucrose 150 g/L, MgSO_4_ 0.5 g/L, agar 20 g/L), at the different time points (I, II and III). The strains were inoculated on 90 mm diameter Petri dishes and incubated at 25 °C for 7 days in the dark. The extraction methodology described in [39] was employed. Briefly, 3 agar plugs were removed from one colony and placed into a 4 mL vial, where 1 mL of methanol was added. After 60 min, the extract was filtered through 25 mm syringe filters with 0.45 μm PTFE membranes (VWR International) into new 2 mL vials, evaporated and further dissolved in 1 mL of a solution composed of water:acetonitrile:acetic acid (99:99:2 v/v/v).

Samples were analysed by HPLC with a Jasco (Tokyo, Japan) FP-920 fluorescence detector (333 nm excitation wavelength; 460 nm emission wavelength). Chromatographic separations were performed on a reverse phase C18 column YMC-Pack ODS-AQ (250 × 4.6 mm, 5 μm; YMC Europa GmbH, Dinslaken, Germany) fitted with a pre-column with the same stationary phase. The mobile phase was water:acetonitrile:acetic acid (99:99:2, v/v) pumped at 0.8 mL/min. The injection volume was 50 μL. OTA standard was supplied by Biopure (Getzersdorf, Austria). An initial calibration curve was prepared, and quantification was made using Galaxie software (1.9.302.952 version) and extracts, with the same retention time as OTA standard, were considered to be OTA positive. The limits of detection (LOD) and quantification (LOQ) were 0.72 ng/mL and 3.29 ng/mL, respectively.

Strains were also tested for the fumonisin B2 (FUM B2) mycotoxin, as previously described [40]. Briefly, strains were inoculated on CYA in 90 mm diameter Petri plates and incubated at 25 °C for 7 days in the dark. For the extraction and derivatisation methodology, 5 agar plugs from one colony were placed into a 4 mL borosilicate amber vial, where 1 mL of methanol:water (75:25, v/v) was added. After sonication for 50 min, the extract was filtered through 25 mm syringe filters with 0.45 μm PTFE membranes (VWR International), evaporated, kept at 4 °C until further derivatisation for FUM B2 detection [40].

Samples were analysed by HPLC with a Jasco FP-920 fluorescence detector (420 nm excitation wavelength; 500 nm emission wavelength). Chromatographic separations were performed on a reverse phase C18 column YMC-Pack ODS-AQ (250 × 4.6 mm, 5 μm), fitted with a precolumn with the same stationary phase. The mobile phase was acetonitrile:water:acetic acid (60:40:1, v/v/v) pumped at 1.0 mL/min. The injection volume was 50 μL. FUM B2 standard was supplied by Sigma-Aldrich (St. Louis, MO, USA). An initial calibration curve was prepared, and quantification was made using Galaxie software (1.9.302.952 version) and extracts with the same retention time as FUM B2 standard were considered to be FUM B2 positive. The LOD and LOQ were 75 ng/mL and 301.4 ng/mL, respectively.

### 2.6. MALDI-TOF MS: Proteome Fingerprinting

Samples at the different timing of preservation (I, II and II) were grown on MEA, incubated in the dark at 25 °C for 4 days and used for spectral analysis by Matrix-Assisted Laser Desorption⁄Ionisation Time-Of-Flight Mass Spectrometry (MALDI-TOF MS) in the range from 2 to 20 kDa. The procedures were followed as described in [23]. Briefly, for the flex target plate preparation, approximately 1 µg of spores and young mycelium of each species was transferred directly from the culture plate to the MALDI-TOF sample plate. Immediately after, 0.5 µL of matrix solution (7.5% 2,5-dihydroxybenzoic acid in ethanol/water/acetonitrile (1:1:1) with 0.03% trifluoroacetic acid) was added to the samples and mixed gently. The sample mixtures were air-dried at room temperature. Each sample was processed in duplicate to test reproducibility. During the analyses, all solutions were prepared and stored at 4 °C.

### 2.7. Molecular Biology: Typing Assays

Genomic DNA was extracted using a modified protocol described in [12]. Briefly, spores of each strain were transferred from a 7 days old culture into 50 mL Falcon tubes containing 25 mL of MGYP. Samples were incubated at room temperature for 5 days in the dark, at 150 rpm in a shaker. Fungal biomasses were filtrated and stored at −20 °C.

For DNA extraction, 100 mg of biomasses were transferred into a 1.5 mL Eppendorf tube containing 100 µL of lysis buffer (200 mM Tris-HCl pH 8.5, 250 mM NaCl, 25 mM EDTA, 0.5% (w/v) SDS). Cell lysis was performed using a pellet pestle for 3 to 4 min. After mechanical lysis, 900 µL of lysis buffer was added, and the samples were incubated for 1 h at 65 °C. Samples were centrifuged at 14,000× *g* for 10 min at room temperature, and 800 µL of the upper phase was transferred into a new 2 mL Eppendorf tube. Polysaccharides and proteins were precipitated by adding 1 mL of cold sodium acetate (3 M, pH 5.5).

Samples were gently mixed by inversion, placed at −20 °C for 10 min and centrifuged at 14,000× *g* for 10 min at room temperature. Clean supernatant was then transferred to a new tube and precipitated with one volume of cold isopropanol (−20 °C). Samples were gently mixed by inversion for 2 min, incubated at −20 °C for 2 h and centrifuged at 14,000× *g* for 10 min. DNA pellets were washed twice with 1.0 mL of cold 70% ethanol, centrifuged at 14,000× *g* for 10 min and dried using a SpeedVac Concentrator. DNA samples were suspended on 50 µL of ultra-pure water, treated with 10 mg of RNase A for 1 h at 37 °C and stored at −20 °C. Samples were subjected to quality assessment by quantification of total DNA using the Qubit dsDNA HS Assay Kit with the Qubit^®^ 2.0 Fluorometer (Invitrogen; Thermo Fisher Scientific, Inc., Waltham, MA, USA), and by electrophoresis on 1% (w/v) agarose gels in Tris-acetate-EDTA (TAE) buffer.

DNA fingerprinting was performed using the M13 phage probe 5′-GAGGGTGGCGGTTCT-3′ [41]. M13 PCR reactions used 1x VWR Taq DNA Polymerase Master Mix with 1 mM MgCl_2_, 0.8 µM primer M13 and 50 ng of genomic DNA in a 25 µL reaction volume. Thermal cycling parameters were 94 °C for 2 min, 40 cycles of 94 °C for 20 s, 50 °C for 1 min, 72 °C for 20 s and a final extension at 72 °C for 6 min. Products were separated by electrophoresis on 1.5% (w/v) agarose gels in TAE buffer.

### 2.8. Data Analysis

For proteome fingerprinting, the data were analysed based on a matrix of pairwise correlation values for spectra after smoothing baseline corrections and peak detections. The peak lists of the selected strains were directly exported to the SARAMIS™ software package (Spectral Archiving and Microbial Identification System, AnagnosTec, Version 2010, Potsdam, Germany, 2010). A dendrogram of proteomic profile proximity among isolates was created using SARAMIS™ package.

For M13 fingerprinting analysis, BioNumerics 7.6 software (Applied Maths NV, Sint-Martens-Latem, Belgium) was used to calculate pairwise similarity values based on the Dice coefficient and converted into a dendrogram by unweighted pair group method with arithmetic means (UPGMA). The cophenetic correlation coefficient was determined, and the values associated with the branches were figured out on the nodes. Additionally, principal component analysis (PCA) was performed to show the relationships among samples.

The experimental design did not involve replication of the experiments, and as a matter of consequence, statistical analysis was not performed. The exception was the work developed for M13 typing assay that was performed in two independent assays. However, the results were similar and only one set of the results are presented.

## 3. Results and Discussion

### 3.1. Viability, Growth and Morphological Analyses

All the twelve selected Aspergillus strains were able to grow on a variety of fungal media after freeze-drying and accelerated storage conditions (37 °C in the dark for 2 and 4 weeks) including MEA, PDA, CZ and CYA. All tested strains successfully survived preservation conditions equivalent to 40-years of storage at 5 °C.

**Figure 1:**
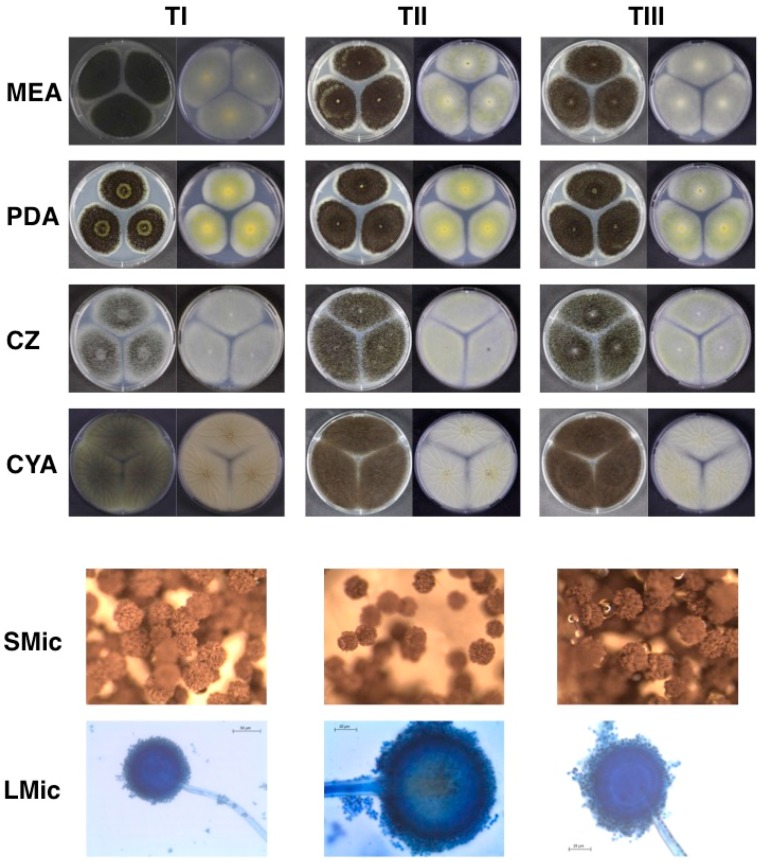
Morphological characteristics of *Aspergillus niger* Filamentous Fungal Culture Collection Micoteca da Universidade do Minho (MUM) 05.11 before (TI), after 2 weeks (TII) and 4 weeks (TIII) of freeze-drying and accelerated storage. Obverse (left) and reverse (right) of colonies grown for 7 days in the dark, at 25 °C on malt extract agar (MEA), potato dextrose agar (PDA), Czapek agar (CZ) and Czapek agar with yeast extract (CYA) media are shown. Stereomicroscope images (SMic) showing general colour and morphological characteristics of colony and light microscopy images (LMic) of conidiophores at the three different ageing times are also presented.

**Figure 2:**
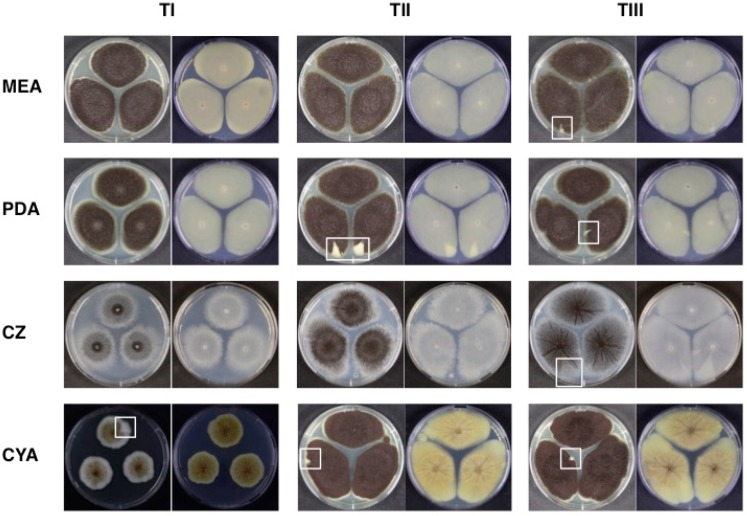
Morphological characteristics of *Aspergillus aculeatus* MUM 03.11 before (Time I), after 2 weeks (Time II) and 4 weeks (Time III) of freeze-drying and accelerated storage. Obverse (left) and reverse (right) of colonies grown for 7 days in the dark, at 25 °C on MEA, PDA, CZ and CYA media. Sectorisation areas are indicated by white boxes.

Figure 1 is a representative image for both the macro-morphological and the micro-morphological characteristics, showing fungal colony general colour and its morphological characteristics, and conidiophores at the three different ageing times under light microscopy. Macro-morphology of the colonies (conidial colour and mycelial colour) showed no substantial differences in the recovered cultures grown in different media and after freeze-drying (Figure 1). In contrast, sectorisation representing phenotypical degeneration was observed in approximately 30% of the inoculated plates. *A. aculetaus* MUM 03.11 (Figure 2) with *A. ibericus* MUM 04.86 were the most sectorised strains observed. When comparing the different preservation time points, we have observed an increased number of sectorised strains for all studied media from points I (9%) and II (9%) to point III (13%) (Table 2). Previous studies have linked this phenomenon to gene expression disruption, unstable mtDNA and mitochondrial dysfunctions, and oxidative stress, having a possible impact in the strains’ capacity to produce secondary metabolites and enzymes [42,43,44,45]. Conidiophores and spores maintained their morphological structure and did not present any changes during and after ageing. Mycelial extension rates of all strains were nearly unaffected by the freeze-drying process, but it was possible to observe a stimulation of conidiophore production and sporulation, particularly in CZ and CYA. No differences in micro-morphological characteristics (presence or absence of clamp connections, presence or absence of hyphal vacuolisation and shape and branching of hyphae) were observed between strains before and after preservation (data not shown). *Aspergillus aculeatus*, *A. japonicus* and *A. uvarum* were the only uniseriate species presenting only phialides whilst all the other strains presented metulae and phialides (biseriate). These micro-morphological observations are totally aligned with previous taxonomic reports for Section *Nigri* [46,47]. Moreover, each species presented spores with different characteristics, either in shape, size and ornamentation features. These features were common and equal for all the time points analysed.

### 3.2. Biochemical Characterisation: Enzymes and Mycotoxins Production

Enzyme production is a fungal characteristic, and enzymatic activity can be analysed as part of any biotechnological potential assessment. To determine the effect of preservation and accelerated storage on the capacity of *Aspergillus* strains to secrete commercially relevant enzymes, all the 12 selected *Aspergillus* strains from the three preservation time points were grown for 7 days either on skim milk agar to analyse proteolytic enzymes activity on day 7 by deep-clearing measurement (Table 3) or on maltose medium and the culture media supernatant from each day was analysed for the production of the polysaccharide-hydrolytic enzymes CMCase, xylanase, pectinase and mannanase at the time points I, II and III (Table 4).

Enzymes analysed in this screening were present in all of the *Aspergillus* strains tested. For all the analysed samples, it was possible to observe that those with more time of accelerated storage presented higher enzymatic activity values, especially when compared with non-aged samples (time point I). Nevertheless, no consistent pattern was observed in the increase of the enzymatic activities.

In addition to its economical relevance, *Aspergillus* section *Nigri* strains represent one of the most important sources of mycotoxins contamination of food and feed [48]. The main mycotoxins produced by this group of filamentous fungi are ochratoxin A (OTA) and fumonisins (FUMs) of the B series, in particular, FUM B2 [49,50,51,52].

For the assessment of OTA and FUM B2, two different concentrations of a standard solution were used as control while injecting mycotoxin samples into the HPLC. No detectable production of FUM B2 was observed for any of the analysed strains (i.e., *A. niger*). The OTA values obtained for each accelerated storage time point are listed in Table 5.

More than 40% of the analysed strains are ochratoxigenic, and some changes in OTA production were found between preservation time points. Although those were apparently not associated with preservation and ageing, results showed that OTA production was stimulated in *A. brasiliensis* MUM 06.179 and *A. vadensis* MUM 06.153 after accelerated storage.

Changes in mycotoxin production patterns due to preservation techniques were observed in other filamentous fungi [53]. However, other authors have shown that the variability in mycotoxins production seems to be more strain-specific than due to the used preservation technique [16,53]. In addition, changes in mycotoxin production patterns have been related to physiological instability in filamentous fungi [54]. Secondary metabolites synthesis is often related to nutrient depletion, abiotic stress, as well as possible ecological roles in nature [55,56].

### 3.3. MALDI-TOF MS Spectral Analysis

When analysing filamentous fungi through MALDI-TOF MS, a signature profile is obtained by pre-processing a raw spectrum and reducing it to a set of m/z and relative intensity points that represent the sample proteome in the range from 2 to 20 kDa [26,28]. Reproducibility of spectra is not always achieved. In fact, a small percentage of differences can be easily justified by variation in several parameters, from culture conditions or sample preparation, to equipment and laser age [27,28]. Furthermore, heterogeneous morphological phenotypes of filamentous fungi can be translated into variable MALDI-TOF mass spectra, either between different strains of the same species as well as between subcultures or distinct tissue samples of the same strain [28,57].

According to Santos et al. [24], fungal identification by MALDI-TOF MS can be impacted by modifications of the protein extraction methodology, and by increasing both the number of reference spectra of a given strain included in the reference library and the number of deposits used to generate each reference spectra. Three recent review works highlight the importance of having a comprehensive and well-curated reference database and how this is still a major limitation for the identification of filamentous fungi using commercially available platforms [26,27,28,29]. However, when using a comparison of spectra to understand the differences between different time points of a preservation method, as in this particular case, the main focus was to observe the main peaks obtained in each spectra with no need to employ any given comparison database.

In all the analysed strains it was possible to see that, after preservation and ageing, there were differences in the spectral data with an increase in the number of peaks, particularly for time point II, or decrease in the case of time point III (Table 6).

**Figure 3:**
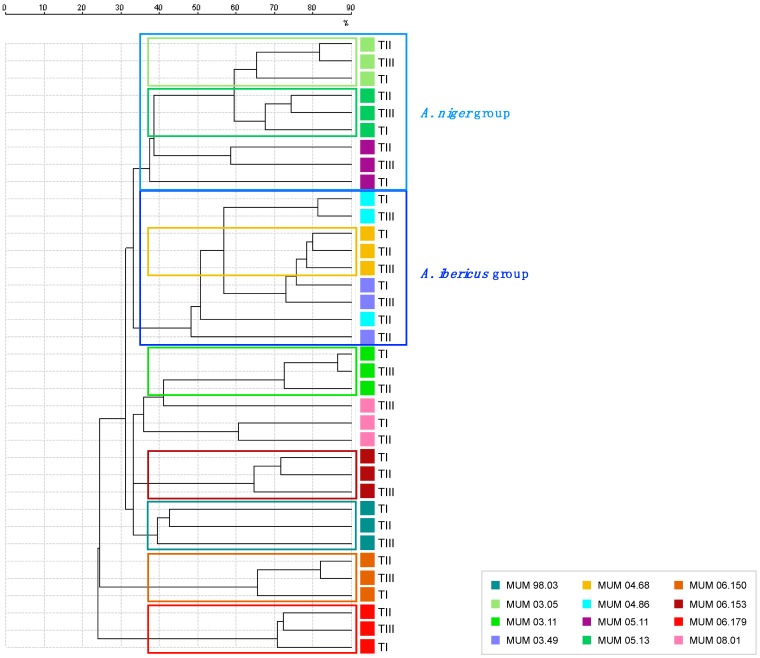
Dendrogram of proteomic relatedness with data from three preservation time points. TI—before preservation; TII—2 weeks of accelerated storage; and TIII—4 weeks of accelerated storage. Coloured boxes indicate strains where the three-time points cluster together.

Such variations were not sufficient to disturb sample grouping in the dendrogram of proteomic profile relatedness presented in Figure 3.

It is possible to observe that 8 out of 12 *Aspergillus* section *Nigri* strains formed groups that included the three-time points tested. Furthermore, for the remaining strains considered here, the three-time points are located in close branches. In other words, the preservation and ageing processes did not affect the proteome in the range from 2 to 20 kDa of the majority of samples and consequently did not significantly affect the grouping of MALDI-TOF MS spectra of fungal strains.

It is also worth mentioning that MALDI-TOF MS spectral analysis is capable of grouping strains within the same species (Figure 3). Our results show that *A. niger* and *A. ibericus* strains form two distinct and well-defined groups. In the case of *A. niger*, our results are in accordance with the recent whole genome based conclusion that *A. phoenicis* is a synonym of *A. niger* [58], as strain MUM 03.05 forms a group at approximately 40% relatedness with MUM 05.11 and MUM 05.13. However, from the MALDI-TOF MS data presented here, we are unable to support the same study’s affirmation that *A. lacticoffeatus* MUM 06.150 is also an *A. niger* synonym which can be related with the metabolic mutation at the PKS gene (*pks*A) for the production of black conidium pigment [59].

### 3.4. Molecular Biology: Typing Assays

**Figure 4:**
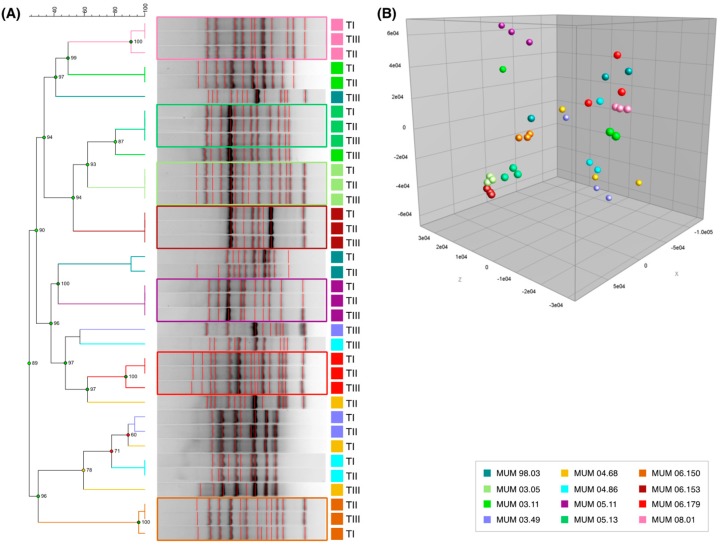
M13 fingerprinting results with data from three preservation time points. TI—before preservation; TII—2 weeks of accelerated storage; and TIII—4 weeks of accelerated storage. (**A**) dendrogram based on Dice coefficient and UPGMA. Cophenetic correlation coefficient values associated with the branches are shown in the nodes. Coloured boxes indicate strains where the three-time points cluster together. (**B**) principal component analysis plot.

To identify genetic changes in strains after preservation, amplification of hypervariable inter-repeat DNA fragments using a single primer M13 was performed. The M13 profiles varied between 0.3 and 3.0 kb with some strains showing a large number of bands with variable degrees of intensity (Figure 4A). This may be related to the number of regions homologous to the specific sequences of M13 dispersed in the genome of each strain [41,60]. Seven out of 12 fungal strains presented little differences in their fingerprinting patterns resulting in clustering of the three time points for each sample: *A. phoenicis* MUM 03.05, *A. niger* MUM 05.11 and MUM 05.13, *A. lacticoffeatus* MUM 06.150, *A. vadensis* MUM 06.153, *A. brasiliensis* MUM 06.179 and *A. uvarum* MUM 08.01 (Figure 4A). The PCA analysis plotted in Figure 4B allows a better visualisation of these relationships where *A. brasiliensis* MUM 06.179 shows more dispersion, immediately followed by *A. niger* MUM 05.11 when compared with the other five strains. It is interesting to note that five of these samples also grouped in the MALDI-TOF MS results.

Recently, a study on *Aspergillus* section *Nigri* has shown a high degree of homogeneity of the species pan-genomes [58]. Nevertheless, inter- and intraspecific variation exists and can be explored in studies involving related strains. A commonly used technique is randomly amplified polymorphic DNA (RAPD) fingerprinting. RAPD can generate highly informative data [61] despite the reported low reproducibility and limitations that include a lack of consideration of possible polymorphisms present in the sequence of equally sized bands [62,63]. Such limitations probably explain the differences in clustering capacity of same species profiles when compared to MALDI-TOF MS (Figure 3). In fact, M13 fingerprinting results (Figure 4) show very limited clustering capacity of *A. niger* strains. As mentioned above, *A. phoenicis* is a synonym of *A. niger* [59], a finding that is only partially supported by our fingerprinting results as the three time points of strain MUM 03.05 cluster with all MUM 05.13 samples. Nevertheless, *A. niger* MUM 05.11 and *A. lacticoffeatus* MUM 06.150 (also defined as *A. niger* synonym by [58]) both cluster in different branches of the dendrogram (Figure 4).

The found genetic polymorphisms can be interpreted as a sign of stress induced by the freeze-drying and ageing processes. These can be related to phenotypical alterations even when the proteome is unaffected as exemplified by *A. aculeatus* MUM 03.11. This strain’s time III fingerprinting profile does not cluster with time I and II and is sectorised after 4 weeks of ageing in all media (Figure 2). Susceptibility to genetic alterations appears to be species-specific—contrary to all *A. niger* strains (including MUM 03.05 and MUM 06.150), each of the three *A. ibericus* strains included in this study show differences between preservation time points (Figure 4). Similar results have been reported [16,20], where genetic polymorphisms were observed after preserving fungal strains by the freeze-drying method, demonstrating that it is necessary to perform long-term validation studies of the used preservation methods.

## 4. Conclusions

In this work, 12 selected *Aspergillus* section *Nigri* strains were preserved by freeze-drying and then aged by accelerated storage in the dark at 37 °C for specific time points. The evaluation of all samples within the several time points was done through a polyphasic characterisation. The methods used showed that with time, the freeze-dried samples suffer changes to variable degrees. Little morphological differences were observed, and those were restricted to sectorisations at macro-morphological level, which can be explained by preservation-induced stress.

Regarding the proteolytic and polysaccharide-hydrolytic enzymes studied here, increased activity was observed, which does not represent a limitation on the strains’ biotechnological potential. On the other hand, the preservation and long-term storage of strains can cause physiological instability that affects mycotoxins production. This effect might be species or even strain-dependent as production was either stimulated or decreased. Furthermore, these conclusions are only based on OTA production levels and in a limited set of strains and so other mycotoxins and species should be further studied.

MALDI-TOF MS results showed that, at a proteomic fingerprint level, strains and species are quite stable. As the proteome, in the range of 2 to 20 kDa, is mainly completed by the fungal ribosomal proteins’ machinery, this technique gives good indications of the low impact that preservation and the long-term store might have on these target proteins. These results also support the idea that MALDI-TOF MS is a valuable tool capable of grouping related samples even when molecular biology tests fail to do so. In addition, and in what M13 fingerprinting is concerned, despite the limitations on the extent of clustering capability of this tool, the observed genetic polymorphisms show some overlap with those of spectral analysis.

In spite of the changes detected using an array of methodologies, we consider that those could not cause major impacts in biotechnologically relevant strains of *Aspergillus* section *Nigri* if the features that make them valuable or unique after the freeze-drying persisted intact. For each valuable or unique strain, the freeze-drying long-term preservation needs to be assessed before having full trust in this technique. In case freeze-drying shows a variation in the uniqueness biotechnological feature that undermines the fit-for-propose of the strain other preservation techniques, such as cryopreservation at −80 °C or under liquid nitrogen, should be considered.

## Figures and Tables

**Table 1:** Selected *Aspergillus* section *Nigri* strains and their associated information.

Species	Strain MUM No.	Geographical Origin	Substrate
*A. aculeatus*	03.11 ^T^ (NRRL 5095 ^T^) *	Unknown	Tropical Soil
*A. brasiliensis*	06.179	Portugal	Grapes Cabernet Sauvignon
*A. ibericus*	03.49	Portugal	Grapes Periquita
	04.68	Portugal	Grapes Aragonês
	04.86	Portugal	Grapes Tinta Barroca
*A. japonicus*	98.03 (DSM 2345) **	Unknown	Unknown
*A. lacticoffeatus*	06.150 ^T^ (CBS 101883 ^T^) ***	Indonesia	Coffee Robusta (Rubiaceae)
*A. niger*	05.11	Portugal	Grapes Periquita
	05.13	Portugal	Grapes Aragonês
*A. phoenicis*	03.05 (NRRL 365) *	Unknown	Unknown
*A. uvarum*	08.01	Portugal	Grapes Tinta Barroca
*A. vadensis*	06.153 ^T^ (CBS 113365 ^T^) ***	Unknown	Dead plant tissue

T = ex-Type strain; * Northern Regional Research Laboratory (NRRL, Beltsville, Maryland, USA); ** Deutsche Sammlung von Mikroorganismen und Zellkulturen-DSMZ (Braunschweig, Germany); *** Westerdijk Fungal Biodiversity Institute-CBS (Utrecht, The Netherlands).

**Table 2:** Percentage of phenotypical sectorisation observed of *Aspergillus* strains grown in four different media after 7 days in the dark, at 25 °C, for different preservation time points (before (Time I), after 2 weeks (Time II) and 4 weeks (Time III) of freeze-drying).

Species	Strain MUM No.	Phenotypical Sectorisation (%)		
		I	II	III
*A. aculeatus*	03.11	25	50	100
*A. brasiliensis*	06.179	25	nill	nill
*A. ibericus*	03.49	50	nill	50
	04.68	nill	50	25
	04.86	50	50	75
*A. japonicus*	98.03	25	50	75
*A. lacticoffeatus*	06.150	50	50	50
*A. niger*	05.11	nill	nill	nill
	05.13	nill	nill	nill
*A. phoenicis*	03.05	25	nill	nill
*A. uvarum*	08.01	25	50	75
*A. vadensis*	06.153	25	nill	25

**Table 3:** Proteolytic activity at day 7 of *Aspergillus* strains different preservation time points (before (Time I), after 2 weeks (Time II) and 4 weeks (Time III) of freeze-drying).

Species	Strain MUM No.	Deep-Clearing Distance (mm)		
		I	II	III
*A. aculeatus*	03.11	<6	11	11
*A. brasiliensis*	06.179	11	11	9
*A. ibericus*	03.49	<6	16	16
	04.68	15	16	14
	04.86	<6	11	14
*A. japonicus*	98.03	8	6	7
*A. lacticoffeatus*	06.150	<6	14	14
*A. niger*	05.11	<6	11	11
	05.13	<6	11	11
*A. phoenicis*	03.05	8	6	11
*A. uvarum*	08.01	<6	11	8
*A. vadensis*	06.153	<6	<6	<6

**Table 4:** Maximum polysaccharide-enzymatic activity detected during 7 days for supernatants of *Aspergillus* strains grown in maltose broth medium at different preservation time points (before (Time I), after 2 weeks (Time II) and 4 weeks (Time III) of freeze-drying).

Species	Strain MUM No.	Maximum activity (nkat/mL)											
		CMCase			Xylanase			Pectinase			Mannanase		
		I	II	III	I	II	III	I	II	III	I	II	III
*A. aculeatus*	03.11	0.018	0.018	0.017	0.011	0.028	0.010	0.056	0.055	0.040	0.031	0.031	0.044
*A. brasiliensis*	06.179	0.018	0.018	0.018	0.015	0.026	0.017	0.038	0.086	0.070	0.044	0.044	0.043
*A. ibericus*	03.49	0.024	0.017	0.016	0.012	0.014	0.011	0.042	0.088	0.038	0.042	0.041	0.042
	04.68	0.018	0.006	0.019	0.013	0.006	0.003	0.042	0.137	0.044	0.032	0.017	0.034
	04.86	0.020	0.021	0.021	0.037	0.035	0.012	0.036	0.080	0.039	0.033	0.034	0.050
*A. japonicus*	98.03	0.016	0.024	0.019	0.006	0.012	0.026	0.048	0.046	0.093	0.038	0.046	0.045
*A. lacticoffeatus*	06.150	-	-	0.006	-	-	0.012	0.016	0.015	0.109	0..26	-	0.029
*A. niger*	05.11	0.017	0.016	0.020	0.014	0.009	0.014	0.026	0.096	0.058	0.043	0.041	0.044
	05.13	-	0.012	0.019	0.008	0.007	0.017	0.026	0.109	0.037	0.016	0.038	0.043
*A. phoenicis*	03.05	0.018	0.024	0.021	0.020	0.015	0.019	0.046	0.041	0.036	0.032	0.034	0.034
*A. uvarum*	08.01	-	0.018	0.022	-	0.01	0.025	0.026	0.038	0.119	0.023	0.046	0.046
*A. vadensis*	06.153	0.014	0.013	0.022	0.008	0.007	0.023	0.036	0.059	0.074	0.039	0.038	0.056

- = no detectable activity.

**Table 5:** Concentrations of ochratoxin A (OTA) determined for 12 *Aspergillus* strains at different preservation time points (before (Time I), after 2 weeks (Time II) and 4 weeks (Time III) of freeze-drying).

Species	Strain MUM No.	OTA ng/mL (Rt * = 16.2 min)		
		I	II	III
*A. aculeatus*	03.11	-	-	-
*A. brasiliensis*	06.179	1.8	17.5	26.8
*A. ibericus*	03.49	-	-	-
	04.68	-	-	-
	04.86	-	-	-
*A. japonicus*	98.03	-	-	-
*A. lacticoffeatus*	06.150	535.5	2.6	705.1
*A. niger*	05.11	2508.0	4858.4	2673.1
	05.13	-	-	-
*A. phoenicis*	03.05	4.2	13.3	7.2
*A. uvarum*	08.01	-	-	-
*A. vadensis*	06.153	-	12.3	39.0

* Rt = retention time; - = no detectable production.

**Table 6:** Variation in the number of spectral data peaks for time points II and III in comparison with time point I for 12 *Aspergillus* strains.

Species	Strain MUM No.	No. of Peaks Variation for Time Points		
		I	II	III
*A. aculeatus*	03.11	75	59	58
*A. brasiliensis*	06.179	91	120	119
*A. ibericus*	03.49	84	87	58
	04.68	65	94	37
	04.86	80	93	48
*A. japonicus*	98.03	54	73	53
*A. lacticoffeatus*	06.150	87	94	97
*A. niger*	05.11	56	69	41
	05.13	79	74	39
*A. phoenicis*	03.05	83	102	66
*A. uvarum*	08.01	106	76	39
*A. vadensis*	06.153	102	99	105
